# On the Transmission of Hereditary Qualities of Mind

**Published:** 1848-10-01

**Authors:** 


ART. VIII.—*Facts and Arguments on the Transmission of Intellectual and Moral Qualities from Parents to their Offspring.* New York. Wiley and Putnam. 1847.

A TRANS-ATLANTIC lady, deeply imbued with kind maternal feelings, and a desire of upholding the dignity of female American society—which she intimates (page 150) surpasses, in domestic comfort, happiness, and purity, any other in Europe—has given to the world, in the volume under the above title, the result of what she terms a life-work of reflection, grounded upon induction from history, observation, and experience, in order to establish the simple proposition that the intellectual and moral qualities of mankind are transmitted from parents to their children; and with the view of substantiating this beyond the possibility of any reasonable disputation, she has recourse to a variety of facts, biographical and historical, and proofs and arguments, direct, indirect, and collateral, which are collectively jumbled together in so amusing a manner as to excite our risible rather than our cogitative faculties. The inquiry itself is, we admit, an important one, which should be conducted with philosophical gravity; but how is it possible to deal harshly with an anonymous lady in America? We cannot do so. We have too much of the milk of human kindness, probably derived from a Castalian spring belonging to one of our remotest great grandmothers, even to attempt it. Nay, we cannot summon up our courage even to read her a lecture on the meaning of the words "philosophical induction," or on the elementary principles of logic. We shall content ourselves with stating the doctrines she promulgates as nearly as possible in her own language, and the reasonings by which she endeavours to support them, interjaculating only an occasional reflection, which may sober down any unbecoming merriment on the part of our readers, for these are matters which should be discussed with a serious bearing, if not with academical dignity. It is the conviction of our authoress, that men of ardent minds and high intellectual capacity derive the prerogatives of their genius exclusively from their mother, and in confirmation of this opinion she cites the illustrious example of Napoleon. The authoress must here speak for herself—

"In the life of Napoleon we learn that his mother, previous to his birth, sharing the fortunes of war with her husband, in constant peril and danger, passed much of her time on horseback. Any person accustomed to this mode of riding, must acknowledge that it causes exciting and aspiring emotions. What conveys to the mind of man greater consciousness of power, than to be raised, as it were, above earth, and direct at will an animal so much his superior in physical strength? There we can have the causes that produced a mind like Napoleon's. The active and health-inspiring habits of his mother gave him a strong constitution, and great physical powers of endurance, while the excitement induced by constant exposure [on horseback] to peril and danger, conduced to an activity of intellect highly favourable in producing corresponding qualities in the mind of her unborn child. And behold the first manifestations of the young Napoleon were pride, an indomitable spirit, a passion for warlike pursuits. These being innate and constantly exercised, increased to such a degree, that nothing short of the subjugation of a world could bound his ambition."—pp. 17, 18.

We confess that we do not exactly perceive the relation here between cause and effect. The mother of Napoleon on horseback, going at a goodish pace, say a hard canter, suggests the picture of a healthful, active-minded woman, albeit, harmonising little with the exquisite statue of her by Canova—but physiologically considered, we do not comprehend how the intellectual qualities of the mother became, by this agreeable equestrian exercise, impressed on the "mind of her unborn child." Neither can we trace any connexion, psychologically, between the embryo as it must then have existed, and the fully developed hero on the plains of Marengo and Austerlitz. Our authoress next adduces the example of Goëthe, and here she shall again state her own case; albeit, the evidence upon which she relies is extracted from Falk's life of this illustrious poet and philosopher :—

"In Goëthe's character we find a most sensitive shrinking from all intense impressions, which by every means, and under every circumstance of life, he sought to ward off from himself. We find the same peculiarity in his mother, as we shall see from the following curious and characteristic traits. They were related to me by a female friend who was extremely intimate with her at Frankfort. Goëthe's mother, whenever she hired a servant, used to make the following condition:—'You are not to tell me anything horrible, afflicting, or agitating, whether it happened in my own house, in the town, or in the neighbourhood. I desire, once for all, that I may hear nothing of the kind. If it concerns me, I shall know it soon enough; if it does not concern me, I have nothing whatever to do with it. Even if there should be a fire in the street in which I live, I am to know nothing of it, until it is absolutely necessary I should.'"—pp. 21, 22.

Here we cannot help protesting that she must have been a very cross, crabbed, uncharitable old lady. We should not like to have lived next door to her. That a servant should be snubbed for letting her know that her neighbour's house was on fire, or scolded for asking permission to carry a bucket of cold water to help to extinguish it, was the extreme of unkindness; "and yet," adds Falk, speaking of the amiable-minded Goëthe, "this was the very blood which flowed from her veins into his." We marvel that any likeness should be sought between such a portraiture and his gentle and philanthropic spirit. We repudiate theory and fact together; neither carries with it conviction. The authoress, however, will have the hereditary stream derived from its maternal source, however darkened it may become in after-years. "The disposition of evil," quoth she, "is inherited, as well as of good, from the parents;" and in confirmation of the doctrine, she somewhat exultingly exclaims, that no unprejudiced person, who is acquainted with the history and character of his parents, can doubt that this was the case with Lord Byron; and she forthwith refers to the well-known irascibility of his mother, somewhat ludicrously quoting a passage from "Childe Harold," in which the noble bard describes himself to have been "nurtured in convulsions." We believe it is quite true that the dowager Lady Byron did allow her organ of combativeness occasionally to get the better of her benevolence, and did incontinently fling the poker and tongs on one occasion at the head of her wilful and wayward son; but we do not believe that the desperate misanthropy of Manfred, or the infidel murmurings of Cain, derived their inspiration from, or were the consequence of, any peculiar physical idiosyncrasy either of his father or mother, for were it so, genius itself would be only the pitiful heirloom of an hereditary constitution, derived from our common parentage. Earth-born in itself, its aspirations would be of the earth—earthy. It is not for us, by any physiological or psychological reasonings, to calculate upon and unravel this mystery. As Wordsworth finely observes—

> " The soul that rises with us, our life's star,
> Hath had elsewhere its setting
> And cometh from afar."

This much, however, we learn from the history of the world, that men of extraordinary capacity do arise, endowed with preternatural vigour of mind, who do anticipate, as it were by inspiration, truths which are not as yet revealed to all mankind. They live in advance of the age in which they are born. They are the prophets of the olden time; they pave the way for the great progress of mankind on the broad highway of humanity, and to attempt to calculate their advent by any ordinary observations upon our common birth and parentage, would be as absurd as to speculate upon the material origin of the stars that shine above us. Such men were Newton, Shakespeare, Goëthe, and it may be, in a lesser degree, Byron; but to speculate upon the transmission of their intellectual capacities upon such narrow principles as our authoress propounds, would be indeed to degrade genius to the level of the physical laws, over which it triumphs.

Having disposed of the evidence derived from the biography of literary men, our authoress next appeals to history, and, unhappily enough for her argument, cites the lives of Catherine de Medicis—" the talented, the profligate, the cruel"—and her vicious sons, who are "forcible examples," she remarks, " of the hereditary descent of vice." The example itself overthrows the hypothesis. But, be it observed, that the state of society in France—we may rather say, throughout Europe—when Catherine de Medicis arbitrated between and governed the most sanguinary factions that ever divided an empire; the political civil wars which, under the pretext of religion, with royalty on one hand, and Hugonotism on the other, disorganised the fabric of civilization itself, and goaded the very passions it roused into existence even to madness. These, and a variety of other causes as ostensible, are overlooked, and the concentrated vice and cruelty of the age thrown upon Catherine de Medicis. But if our authoress will go a little deeper—if she will consult Mezeray, le Laboureur, De Thou, or the Jesuit, Father Daniel—she will find they all concur in describing Catherine de Medicis as a woman of the most extraordinary intellectual capacity—" C'est un esprit vaste et profonde," says the president Henault, "un âme firme et indomptable, et qui, malgré sa raideur, sait se plier et prendre toutes les formes qui lui sont utiles." Nay, if she will proceed further, and examine the events of the day, as recorded by Tavannes, Castelnau, Brantôme, Mathieu, and the contemporary memoir-writers of that period, she will find that the crimes ascribed to Catherine de Medicis are not only apocryphal, but that there is no satisfactory evidence of their commission. But the point which bears more immediately on the present argument is, that the sons of Catherine de Medicis, Charles IX., Henry III., Francois II., were all men, if not of weak, of very mediocre intellect; and how happens it, if the intellectual and moral qualities of the mother are so clearly transmissible to her offspring, that all vestige of the great abilities which had so long thrown a lustre over the house of the Medici family should have expired with Catherine de Medicis, and not have been inherited by any one of her sons? Other examples in history are not wanting to disprove the hypothesis, either that illustrious women give birth to men of superior intellect, or that great men are invariably born of able women. The authoress puzzles herself to account for the son of Madame de Stael being intellectually so unworthy of his highly gifted mother; but, in contradiction to her theory, she might have cited a more notable and irreconcileable example in the person of Charles V. The mother of the emperor, Charles V., Jeane of Castille and Arragon, was a woman of weak mind, and died an imbecile, insane from imbecility of intellect.

One of the greatest errors which persons sanguine in the pursuit of scientific discovery are apt to commit is that of generalizing and deducting important conclusions from an insufficiency of facts. This is the rock upon which our authoress splits. In the demonstrative sciences the mathematical blunderer is instantly detected; but in the more speculative regions of physiology and psychology the facts themselves require to be sternly analysed. They often assume the appearance of truth, when they are only *ignes fatui*, that gleam across our path only to deceive us. Under the fictitious notion that she is making an induction, by citing a few very equivocal facts, our authoress leaps at many remote conclusions. Among other crotchets which she propounds with an air of philosophy, is the following:—

"The first children of very young mothers, whatever sprightliness they may evince from a high flow of animal spirits, are generally deficient in strength of intellect and stability of character." "It may, indeed, be observed that men most conspicuous for native strength of mind, were not generally the first-born of their parents. Dr. Franklin was the fifteenth child of his father, and the eighth of his mother. Benjamin West was the tenth child of his mother. The mother of Dr. Samuel Johnson was past forty at the period of his birth, and the mother of Washington was twenty years of age when her illustrious son was born."

In the life of women, the interval between twenty-eight and forty years of age affords rather a wide range of latitude for the birth of children of ordinary, as well as extraordinary capacities; but the authoress is an American, and doubtless holds the laws of primogeniture in abhorrence.

The influence of the imagination of the mother upon the mental, as well as physical character of her future offspring, is, of course, an essential part of her creed. There cannot be a doubt, she remarks, on the part of any unprejudiced person who will take the trouble of reading the notes to Hume's History of England, that,—

"The strong sensuality in James must be ascribed to both his parents, whilst his partial idiocy and nervous trembling were, doubtless, caused by the terror which his mother experienced at the brutal murder of Rizzio."—pp. 35, 36.

Our readers may also remember that this feeble monarch manifested a very singular antipathy to the sight of a pig, and we may as reasonably infer that his mother, when she was supping at Holyrood, with her sister, the Countess of Argyle, upon the eventful night of the murder, partook of a dish of fricasseed pettitoes, which proved difficult of digestion. Hence the physical idiosyncrasy of James I. caused him to shudder at the sight of a pig, as well as at that of a drawn sword. These and other as recondite facts have been gravely discussed upon this as well as upon the other side of the Atlantic, and were we inclined to break a lance with our fair American, whose volume has amused us, we should enter the arena under the banners of a host of authorities. But the better part of valour is discretion; and harmless credulity ought not to be harshly dealt with, particularly if it carry along with it a good, practical, moral lesson. We will do our authoress the justice to affirm that this is the case, and that she deduces from her theory precepts and advice which every mother may read with satisfaction and follow with advantage; indeed, we cannot close our notice of the volume before us more appropriately than by quoting the following passage, which, with many others of a similar description, conveys sentiments which must command the most cordial approbation:—

"In order to attain a great and good national character, give women attainments rather than accomplishments. Point out to them their capabilities and responsibilities. Let them know that they are responsible for the moral character of the rising generation; and also that it depends upon themselves whether they become the mothers of wise and virtuous, or foolish and vicious men. For in the same degree as these qualities are possessed and exercised by themselves, will our children inherit and practise them."

---

